# Maximizing Regulatory Review Efficiency: The Evolution of the FDA OCE RTOR Pilot

**DOI:** 10.1007/s43441-021-00371-z

**Published:** 2022-01-10

**Authors:** Ya Grace Gao, Samantha Roberts, Allison Guy

**Affiliations:** 1 Product Development Regulatory, Hoffmann-La Roche Ltd, Mississauga, ON Canada; 2 Leslie Dan Faculty of Pharmacy, University of Toronto, Toronto, ON Canada; 3 Product Development Regulatory, Genentech, Washington, DC USA

**Keywords:** Real-time oncology review, US food and drug administration, Oncology drug review, Oncology center of excellence, Expedited review program, FDA OCE pilot programs

## Abstract

To promote the efficient review of oncology drug applications, the US Food and Drug Administration (FDA) Oncology Center of Excellence (OCE) launched the Real-Time Oncology Review (RTOR) pilot program in 2018. RTOR allows FDA to review individual sections of eCTD modules of a drug application for oncology drugs in contrast to requiring the applicant to submit complete modules or the complete application before review is initiated. Initially, the program accepted only supplemental applications with simple study designs and easily interpretable endpoints, but the scope has since been expanded to include applications for New Molecular Entities (NME), and other applications with more complex features. Though many applicants experience faster approvals under RTOR, it is difficult to isolate the effect of the RTOR program on review timelines as its contribution is masked by other expedited programs like priority review and breakthrough therapy designation (BTD). This article discusses the expanded scope of RTOR, its interplay with other OCE initiatives to modernize regulatory review, summarizes Genentech’s experiences in planning RTOR submissions from February 2019 to July 2021, and provides considerations for the future of the program.

## Introduction

In February 2018, FDA’s OCE initiated the RTOR pilot program to facilitate efficient review of oncology drug applications. Traditionally, the assembly and preparation of a drug application package often required several months to complete and FDA review would not start until a complete package was received. Even in cases where expedited review is granted where rolling review is permitted (e.g., for applications with fast track or BTD), applicants are required to submit completed electronic Common Technical Document (eCTD) modules when they are available for FDA review [1]. In contrast, RTOR allows applicants to submit key sections of eCTD modules of an oncology drug application independently of the entire module. This in turn enables FDA to initiate the review process before a complete module or application is received [2]. The earlier review allows FDA to identify issues (if any) with the data quality and standards, as well as to assess any key regulatory questions, earlier than a traditional review. This, in turn, allows the applicant the opportunity to respond before the entire application is received by the Agency and the PDUFA clock starts [3]. Thus, RTOR enhances the efficiency of drug application review, which could enable earlier patient access.

This paper reviews the evolving scope of RTOR over the last three years, such as the inclusion of NME applications, applications with companion diagnostics (CDx) and applications reviewed in parallel under Project Orbis. This paper also summarizes Genentech’s experience with RTOR between February 2019–July 2021 and discusses the potential future direction of RTOR to enhance earlier access to lifesaving medications.

## Original Scope of RTOR

OCE initially set out three general criteria for selecting oncology drug-licensing applications to participate in the RTOR program. First, the drug should be likely to demonstrate substantial improvement over available therapy [3]. By definition, this means that drugs that have previously received BTD or have met the criteria for other expedited review programs may be eligible for RTOR. The second is a straightforward study design. Lastly, the endpoints in the study should be easily interpreted. In the early phase of the program, RTOR was limited to supplemental applications for products with which FDA had prior experience and which had robust safety databases. Submissions with greater complexity (e.g., applications with a companion diagnostic) or where regulatory confidence had not yet been established (e.g., NMEs) were considered for RTOR on a case-by-case basis, though initially not included in the pilot [2].

**Figure 1:**
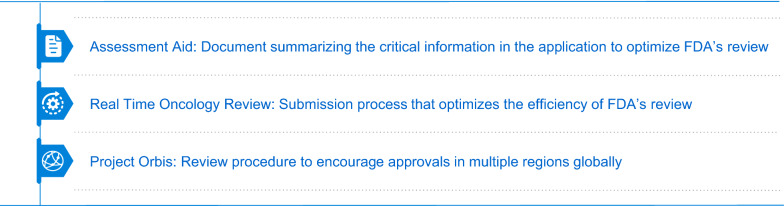
FDA OCE Programs to Modernize Regulatory Review.

Since December 2018, the scope of the pilot program has expanded to include applications for NMEs and applications with more complex features as noted above. Furthermore, RTOR is now being integrated with other OCE pilot programs, such as Project Orbis and the Assessment Aid, all of which aim to modernize regulatory review and make it more efficient [2] (Fig. 1).

## Expanded Scope of RTOR

### NME Applications

In December 2018, the RTOR pilot program began to accept NME applications (Table 1), which are more complex and time consuming to review since they involve new efficacy, safety, and chemistry, manufacturing and controls (CMC) data that have not been previously reviewed by FDA. As of the end of May 2021, there were a total of 10 NME applications approved under RTOR, spanning a wide range of oncologic indications. These applications were based on either phase 3 randomized controlled trials with simple 2-arm study designs or small phase 1/2 studies with single-arm design due to a lack of standard of care in the studied indication. The primary endpoints for these studies included progression free survival (PFS) or overall response rate (ORR), which are easily interpretable. All 10 applications have also received at least one other expedited pathway designations such as priority review and BTD. Hence, although OCE is expanding RTOR to NME applications, the Agency is still adhering closely to its previously set out eligibility criteria.

**Table 1:** New Molecular Entity Applications Accepted Under FDA’s RTOR Pilot^a^

Molecule	Special Designations	ORBIS	Pivotal Trials	Disease Area	Review Time (Months)	Approved	CDx
Piqray (alpelisib)	PR	No	Ph3 randomized, placebo controlled, double blinded; PFS	HR+, HER2−, PIK3CA mutated, adv or mBC	5	2019–05–24	Therascreen PIK3CA RGQ PCR Kit
Tukysa (tucatinib)	BTD ODD PR	Yes	Ph3 randomized, placebo/active controlled, double blind; PFS	2L HER2 + adv unrespectable or mBC (including brain mets)	4	2020–04–17	No
Qinlock (ripretinib)	BTD ODD PR	Yes	Ph3 randomized, placebo controlled, double blinded; PFS	4L Advanced GIST	5	2020–05–15	No
Blenrep (belantamab)	AA BTD ODD PR	No	Ph2, open label, randomized; ORR by IRC	5L Multiple Myeloma	8	2020–08–05	No
Danyelza (naxitamab)	AA BTD ODD PR	No	Ph2 single arm, open label; ORR	2L relapsed or refractory high-risk neuroblastoma	8	2020–11–25	No
Gavreto (pralsetinib)	AA BTD ODD PR	No	Ph1/2 open label, single arm; ORR by BICR	Adv or met RET mutant MTC	5	2020–12–01	No
Tepmetko (tepotinib)	AA BTD ODD PR	Yes	Ph2 single arm, open label; ORR by BICR	NSCLC with MET Exon 14 skipping	7	2021–02–03	No
Jemperli (dostarlimab)	AA BTD PR	No	Ph1 open label, single arm; ORR, DoR by BICR	dMMR recurrent or advanced EC	16^b^	2021–04–22	VENTANA MMR RxDx Panel
Lumakras (sotorasib)	AA FT BTD ODD PR	Yes	Ph1/2 open label, single arm; ORR, DoR by BICR	KRAS G12C-mutated LA or met NSCLC	5.5	2021–05–28	Therascreen KRAS RGQ PCR Kit Guardant360® CDx (plasma)
Truseltiq (infigratinib)	AA FT ODD PR	Yes	Ph2 open label, single arm; ORR, DoR by BICR	2L LA or met cholangiocarcinoma with FGFR2 fusion or other rearrangement	8	2021–05–28	FoundationOne CDx

^a^Up to May 2021

^b^Extended review timeline due to COVID-19-related travel restrictions preventing manufacturing inspection [4]

*AA* Accelerated approval, *BTD* breakthrough therapy designation, *ODD* orphan drug designation, *FT* fast track, *PR* priority review

### Companion Diagnostics

The RTOR program initially did not include applications with a parallel companion diagnostic (CDx) review. However, RTOR has since expanded to accept both supplemental applications and NME applications that include a CDx, the first being the Tibsovo supplemental new drug application (sNDA) in December 2018 using the Abbott RealTime IDH1 CDx. Following Tibsovo’s lead, 7 more applications were accepted under RTOR with contemporaneously developed CDx.

Companion diagnostics (CDx) and the corresponding therapeutic products require applications to be reviewed by Center for Devices and Radiological Health (CDRH) and Center for Drug Evaluation and Research (CDER), respectively, which may lead to different timelines for the approval of the CDx and therapeutic product. Piqray (alpelisib) was the first NME to be accepted for the RTOR pilot and was also one of the first RTOR applications that included a CDx. Despite the increased number of stakeholders involved due to the inclusion of a CDx, and the potentially different review timelines, FDA agreed to standing bi-weekly teleconferences with Novartis, in addition to separate meetings to discuss the CMC section, showing the Agency’s interest in expanding the scope of the RTOR pilot [5]. For these applications, the decision of RTOR eligibility was jointly made between the clinical division director at the CDER, the review team including the reviewers at the CDRH, and OCE management [3].

### Project Orbis

Project Orbis is a pilot launched in 2019 by the OCE at FDA to leverage the existing scientific and regulatory partnerships between international regulatory authorities and provide a framework for concurrent submission and review of marketing applications for high-impact oncology drugs that typically meet the criteria for FDA’s priority review. The aim of the initiative is to facilitate simultaneous and faster patient access to innovative cancer therapies with a high unmet medical need across multiple countries [6]. Sponsors may participate in RTOR and Project Orbis concurrently provided they meet the eligibility criteria for each pilot. The first joint use of both programs was for the approval of Keytruda in combination with Lenvima for the treatment of advanced endometrial carcinoma in September 2019. Since then, 21 out of 31 RTOR applications have also participated in Project Orbis (Table 2). Participating in both pilot programs may introduce added procedural complexities for the applicant in terms of submission planning, communication with and between Health Authorities, and aligning approval timelines to prepare for product launch. This is primarily because the RTOR pilot is a US specific program, whereas Project Orbis involves multiple participating Health Authorities. Other countries participating in the Project Orbis pilot may not have the regulatory flexibility to follow the same submission and review processes as the US [7].

**Table 2:** Comparison of Applications Reviewed Under RTOR and RTOR Combined with Project Orbis Since the Launch of Project Orbis in 2019 to May 2021

Total Applications		Median Approval Time (Range)	Mean Approval Time
RTOR and Project ORBIS	21 (5 NMEs)^a^	4.5 Months (1.5, 8)	4.4 Months
RTOR Alone	10 (4 NMEs)	5 Months (3, 16)	6.5 Months^b^

^a^All received priority review except for nivolumab sBLA

^b^5.4 months if dostarlimab is removed from the calculation

Despite the added challenges of coordinating two pilot programs, there are many advantages such as the FDA sharing its independent analyses of the data with health authorities participating in the review. Furthermore, the average review time for applications that went through both RTOR and Project Orbis is approximately 4.4 months, which is shorter than the 6.5 months mean approval time for applications that participated in RTOR alone. The apparent improvement in the average approval timeline for RTOR and Project Orbis duos could be because there was a much higher proportion of supplemental applications that were based on pivotal studies with straightforward study designs (i.e., large phase 3 randomized controlled trials with simple 2-arm designs).

## Genentech’s Experience with RTOR From February 2019 to July 2021

Genentech first participated in the RTOR program with a supplemental marketing application for Kadcyla’s early breast cancer indication in February 2019 (Table 3). Since then, Genentech has participated 5 more times in the RTOR pilot with 4 supplemental applications and one NME, all spanning a wide range of indications. Among the 6 applications, 3 also participated in Project Orbis. Based on its experience with the program, Genentech has the following recommendations for applicants preparing applications for RTOR.

**Table 3:** Summary of Genentech’s RTOR Experience^a^

Molecule	Type	Special Designations	Indication	Approval Timeline^b^ (Months)	CDx	Project ORBIS
Kadcyla (ado-Trastuzumab emtansine)	sBLA	BTD, PR	HER2 + EBC	3	Ventana PATHWAY anti-HER-2/neu (4B5) Rabbit Monoclonal Primary Antibody assay INFORM HER2 Dual ISH DNA Probe Cocktail assay	No
Venclexta (venetoclax)	sNDA	BTD, ODD, PR	1L CLL	2	N/A	No
Gavreto (pralsetinib)	NDA	AA, BTD, ODD, PR	RET mutation, fusion + LA MTC	5	N/A	No
Venclexta (venetoclax)	sNDA	AA, BTD, ODD, PR	1L Unfit AML	5	N/A	Yes
Tecentriq (atezolizumab)	sBLA	BTD, ODD, PR	1L HCC	4.5	N/A	Yes
Tecentriq (atezolizumab)	sBLA	N/A	Adj NSCLC	Ongoing	N/A	Yes

^a^Up to July 2021

^b^After submission of final RTOR component

*AA* Accelerated approval, *BTD* breakthrough therapy designation, *ODD* orphan drug designation, *PR* priority review

### Communication with FDA

The RTOR pilot involves submission of multiple components at different time points and close collaboration between the Agency and applicants. Therefore, applicants should seek alignment with FDA on the Agency’s expectations for the RTOR process (e.g., communication styles, submission schedule) upon acceptance into the pilot. Different review divisions may have different preferences for the method and frequency of communication. For example, one review division may prefer standing teleconferences, whereas another division may prefer communication via email. Therefore, applicants should engage early with the FDA review division to understand their preferences and expected level of engagement during the RTOR process.

Based on our experience, we have learned that products approved under RTOR can have approval timelines well in advance of their PDUFA goal dates. Hence, applicants should strive to have early and open communication with FDA to understand the review progress and expected approval timeline, if possible, which in turn will help applicants prepare for product launch at the time of approval and ensure timely patient access.

### Resourcing

The preparation for RTOR can be resource intensive especially in the first 12 weeks following study unblinding. Therefore, applicants need to carefully assess whether there are adequate resources to accommodate preparation of the RTOR components outlined in the FDA RTOR Standard Operating Procedures (SOP) [3]. Specifically, those teams working on the datasets and analysis programs, as well as the proposed label, play an important role in RTOR submission preparation, as these components should be submitted early on in the process to enable RTOR review. These teams need to be prepared to work on key deliverables at risk prior to top-line data availability. Both applicants and reviewers may benefit from conversations about hypothetical submission timelines for each component to better support resourcing prior to the availability of the top-line results, particularly for drugs that have qualified for other expedited programs like BTD [8]. Another caveat of participating in RTOR is the reduced time to respond to incoming information requests. Applicants should prepare the filing team for the short turnaround time to respond to FDA requests and ensure the filling team continues to be adequately resourced throughout the submission and review process to address FDA feedback as information requests can come in any time after the initial component is submitted.

### Submission Planning

In order to efficiently manage RTOR activities, applicants should consult the FDA RTOR SOP [3] and clearly map out filing dossier components and delivery timelines as early as possible since oncology review teams at FDA prefer to receive documents as soon as they are finalized. The key deliverables that are critical to ensure an efficient review process are datasets and analysis programs, the draft label, and the Assessment Aid (AAid). Therefore, applicants may benefit from achieving early agreement on the indication statement and alignment between key messages in the clinical documents and key claims in the label early on to allow faster preparation of the draft label once top-line data are available. Also, despite being able to access key components of the dossier earlier than during a typical filing, as FDA conducts its own analyses of the data, the in depth review does not start until the key datasets have been received from the Sponsor. FDA may also request that applicants submit safety update reports and associated datasets sooner than the typical 90-day timeline for priority review. Therefore, applicants may need to accommodate such requests by implementing an earlier data cutoff date for the safety update, anticipating shorter times for data cleaning and output generation, and/or reducing applicant review time for the safety report. Successful utilization of the RTOR pilot will depend on careful planning by applicants to achieve effective submission of these critical deliverables since they may impact the review timeline.

### Assessment Aid

The AAid is another pilot program developed by the OCE to optimize review of oncology product applications and enhance efficiency and consistency. It was developed based on the FDA Multidisciplinary Review document template that summarizes the key aspects of an application that contribute toward the benefit-risk assessment of a drug. All of Genentech's RTOR applications included an AAid, since oncology reviewers prefer it to be used with RTOR to enhance the efficiency of the review process. The expectation from OCE is to have the completed AAid submitted prior to the final component between weeks 6–9 after RTOR is requested [3]. Most of the Genentech RTOR applications submitted AAid as the second to last component. However, not all review divisions have the same flexibility and some may require the AAid much earlier in the process, while others may allow it to be submitted following the submission of the final component. Hence, applicants should also discuss AAid plans with the review division early on, especially if an alternative submission timeline is needed, as not all review divisions have the same amount of flexibility. OCE has provided detailed guidance on the acceptable length and content of the AAid—the total document should be within 100 pages for NME applications and 75 pages for supplemental applications [9]. The Applicant should include only critical information in the AAid, such as information that will appear in the updated label, and avoid promotional language. FDA has stressed that all data in the AAid will be cross-verified with Clinical Study Reports and Module 2 documents. Therefore any extraneous data will add to the review timeline.

## The Impact of RTOR on Oncology Drug Review

The purpose of RTOR is to enable a more efficient review process to ensure that new treatments are available to patients as early as possible [2]. Although shortening review timelines is not an explicit goal of the RTOR program, many applicants have benefited from earlier approvals under the pilot. All 10 NMEs approved under the program also received priority review and the time-to-approval from the submission of the final component ranged from 4 to 16 months with an average of 7.15 months. This is shorter than CDER’s goal of an 8-month review timeline from the time of submission for applications under priority review. Most of the applications were approved before their PDUFA goal date, with Jemperli (dostarlimab) being the only outlier with a 16-month approval timeline (Table 1). The delay in Jemperli’s approval has been attributed to COVID-19-related travel restrictions preventing manufacturing inspection [4]. When Jemperli was removed from the calculation, the average time-to-approval for NME applications reviewed under RTOR was further reduced to 6 months.

A direct comparison of timelines for approval of RTOR vs non-RTOR applications showed that the impact of RTOR on review timelines is likely masked by concurrent use of other expedited programs. In 2020, there were 16 oncology NMEs approved by FDA and among these 5 participated in RTOR. The average approval timeline for the 5 RTOR NMEs was 6 months after submission of the final component, while the average approval time for the 11 non-RTOR oncology NMEs was approximately 6.48 months (Table 4). All 5 RTOR NME applications also received priority review and BTD.

**Table 4:** Comparison of Time-To-Approval Between RTOR and Non-RTOR NME Oncology Applications Approved in 2020

	Total Applications	Median Approval Time (Range)	Mean Approval Time	Applications with Priority Review	Median Approval Time (Range)	Mean Approval Time	Applications with BTD	Median Approval Time (Range)	Mean Approval Time
RTOR NMEs	5	5 months (4, 8)	6 months	5	5 months (4, 8)	6 months	5	5 months (4, 8)	6 months
Non-RTOR NMEs	11	6 months (5.25, 10)	6.48 months	9	6 months (5.25, 7)	6.03 months	5	5.5 months (5.25, 7)	5.85 months

Under PDUFA VI the review timeline for a standard application is 10 months from the 60-day filing date and 6 months for one with priority review (or 12 months and 8 months, respectively, from the date of submission of the application) [10]

*Non-RTOR NMEs include* Darzalex Faspro (daratumumab and hyaluronidase-fihj), Gavreto (pralsetinib), Inqovi (decitabine and cedazuridine), Jelmyto (mitomycin hydrogel), Onureg (azacitidine), Pemazyre (pemigatinib), Phesgo (pertuzumab, trastuzumab, and hyaluronidase-zzxf), Retevmo (selpercatinib), Tabrecta (capmatinib), Tazverik (tazemetostat hydrobromide), Zepzelca (lurbinectedin)

Among the 11 non-RTOR NME oncology applications, 9 had priority review and 5 had BTD. The average time-to-approval was approximately 6.03 months for the 9 non-RTOR NME applications with priority review, and 5.85 months for the non-RTOR applications with BTD, which are comparable to the timeline for those that participated in RTOR. Hence, it is difficult to isolate the contribution of RTOR on review timelines since all RTOR applications also participated in other expedited programs.

Although the impact of RTOR on review timelines is difficult to assess, the program is transforming Industry practices with respect to how applicants prepare applications and engage with reviewers. Since RTOR increases the opportunity for interactions with FDA reviewers throughout the submission and review process, applicants can better understand FDA’s potential concerns and proactively address them before the submission of the final component of the application. Therefore, an issue that may have typically arisen during the review of the application can be raised before a complete application is received, allowing the applicant to incorporate additional information. As such, although RTOR pilot is still a work in progress, it has the potential to ultimately shorten overall review timelines and expedite patient access to effective therapies as both FDA and applicants become accustomed to its procedures.

## Looking to the Future

Though the scope of the RTOR pilot was originally quite narrow, over the past 2 years OCE has expanded the program from supplemental applications with simple study designs to more complex NME applications, including those that include a CDx. Furthermore, FDA has begun to explore more comprehensive ways to assess the benefit-risk profile of new therapies with an increasing emphasis on new sources of data, including real-world data and patient-reported outcomes or other patient experience data [11, 12]. Despite this evolution, the criteria outlined by FDA remain unchanged from when RTOR was first launched and criteria for NME applications or those that contain a CDx is lacking. Hence, OCE should encourage applicants to engage in an early assessment of RTOR eligibility with OCE to allow sufficient time for the applicant to prepare the RTOR submission since considerable resources are needed for the applicants to accommodate the RTOR submission schedule. Applicants should also continue to proactively build their capabilities to scale to accommodate RTOR and stay abreast of the evolving regulatory landscape.

Given the unmet medical need and life-threatening nature of most cancers, oncology drug approvals often benefit from regulatory flexibility in the form of reliance on a single pivotal trial, making them ideal for the RTOR pilot. However, Industry and FDA have recognized that RTOR-type reviews would be beneficial to therapeutic areas outside of oncology, particularly for those products that treat rare diseases as well as for non-oncology products that address serious and unmet needs with large societal impacts. Therefore, as part of the FDA-Industry negotiated PDUFA VII goals, FDA has committed to launch a new pilot program called the Split Real-Time Application Review (STAR), which will allow certain efficacy supplement applications to be “split” into two components for review and has the explicit goal of shortening the time between the receipt of the complete submission by FDA and the action date [13]. While STAR will be distinct from RTOR in terms of eligibility criteria and approach, they both aim to establish an efficient review process, and we look forward to learning more about this program and anticipate that it will enhance patient access to innovative therapies that target a wide variety of conditions.

## Conclusion

OCE has continued to expand the scope of the RTOR program to optimize the regulatory review of oncology treatments. Both reviewers and applicants have gained valuable experience from each new RTOR application and have a better understanding of the process and expectations on both sides with regards to resource allocation and submission planning strategies. The iterative learning has led to enhanced efficiency and expansion of the scope of the RTOR program. We are hopeful that this pilot program can inform the review of therapies beyond oncology products and that it may be used as an example for other countries to adopt similar procedures to improve review efficiency and maximize patient access to innovative medicines globally.
